# Health and Wellness Impacts of Traditional Physical Activity Experiences on Indigenous Youth: A Systematic Review

**DOI:** 10.3390/ijerph17218275

**Published:** 2020-11-09

**Authors:** Lamia Akbar, Aleksandra M. Zuk, Leonard J. S. Tsuji

**Affiliations:** 1 Department of Physical and Environmental Sciences, University of Toronto, Toronto, ON M1C 1A4, Canada; aleksandra.zuk@utoronto.ca (A.M.Z.); leonard.tsuji@utoronto.ca (L.J.S.T.); 2 School of Nursing, Faculty of Health Sciences, Queen’s University, Kingston, ON K7L 3N6, Canada

**Keywords:** traditional physical activity, North America, Oceania, Indigenous, children, youth, holistic approaches

## Abstract

Traditional physical activities have numerous physiological and psychosocial benefits for Indigenous youth around the world. Little is known about the positive health and wellness impacts of traditional physical activity experiences on Indigenous youths. The aim of this systematic review is to explore the holistic health and wellness impacts of traditional physical activities on Indigenous youth from certain North American and Oceania geographic areas. A systematic search of four electronic databases (PubMed, ERIC, Scopus and Web of Science) was conducted to identify peer-reviewed publications of qualitative research exploring the diverse health experiences of traditional physical activities for Indigenous youth in Canada, the United States of America, New Zealand and Australia. A qualitative synthesis of studies between 2006 and 2018 were included, and findings were synthesized using an integrated Indigenous-ecological model, which broadly captures health and wellness impacts under intrapersonal, interpersonal, organizational, community and policy level outcomes using medicine wheel teachings. In total, nine studies were identified via this search. Overall, the literature described numerous emotional, mental and spiritual benefits of traditional physical activity, and youth experiences were affected by familial and communal relationships, and systemic factors. Among Indigenous youth, this research shows the importance of including traditional physical activity in future programs and partnerships with community expertise.

## 1. Introduction

Physical activity through movement and energy expenditure has numerous psychosocial, physiological and developmental benefits for young people [1,2]. A number of studies have specifically assessed the impacts of recreational sports and physical activity on global Indigenous youth, and found them to be holistically beneficial [3,4]. A systematic review on positive youth development (PYD) through physical activity on global Indigenous youth consistently highlighted the need for incorporating culturally relevant physical activity [3]. McHugh, Deal, Blye, Dimler, Halpenny, Sivak and Holt [4] similarly found that connection to culture, traditional sport and recreation were intrinsic aspects defining the physical activity experiences of Indigenous youth within Canada. Traditional physical activities not only include traditional games such as lacrosse, canoeing, and ceremonial dances, but also subsistence activities, such as hunting, fishing, gathering and preparation of foods [5,6]. Furthermore, Indigenous youth tend to understand physical activities to be inclusive of work, household chores, recreation, and cultural activities [7]. Yet, literature, including reviews, specifically studying the complex experiences and impacts of participating in these wide-ranging traditional physical activities is noticeably absent, and research on physical activity in general tends to focus on non-Indigenous, Eurocentric definitions.

Colonialism continues to encroach on how Indigenous populations interact with their cultural and traditional activities today, through legacies of past assimilation, as well as contemporary colonial praxis. In the region now known as Canada, federal policies such as the Indian Act were employed as a paternalistic means to restrict components of Indigenous lives deemed incompatible with Euro-Canadian Christianity [8,9]. This included gatherings and cultural ceremonies, and their replacement with Euro-Canadian sports, which were considered a way to “civilize” [8,9]. Hunting and gathering were further affected by treaty agreements that reduced land access and resources for communities [10]. Similar “anti-Indian” assimilation tactics, including dispossession of land and residential schools, also impacted the cultural practices of Native Americans in the United States [11,12,13]. In Australia, traditional games and sports were intrinsic aspects of pre-colonial Aboriginal identity, a part of daily life [14]. They were especially important for youth as survival skills were taught through these games [14]. Like in North America, traditional rituals, including physical activities, were severely disrupted during European colonization in Australia [14]. For Māori people (New Zealand), similar sanctions and missionary schooling to civilize led to the dissipation of traditional activities among some clans [15]. Beyond the transgenerational legacies of these acts, ongoing colonial practices continue the violation of Indigenous freedom and, ultimately, hinder the lives of Indigenous peoples. This includes continual dispossession of land that prevents traditional and ceremonial practices; thereby, further endangering the livelihood and well-being of Indigenous peoples [16,17,18,19,20]. Indigenous peoples across settler states also face violent racism [21,22,23,24], imprisonment and profiling [25,26,27], and erasure of traditions and languages [16,17].

The United Nations Declaration on the Rights of Indigenous Peoples (UNDRIP), which calls for Indigenous peoples’ rights to maintain and protect traditional games (Article 31), was initially opposed by Australia, Canada, the United States, and New Zealand, and accepted in later years [28]. Since the adoption of UNDRIP, many programs and initiatives have emerged in the field of traditional activities research. For many Indigenous youth today, partaking in traditional physical activities is a form of showing pride and connecting with culture and life on the land [29]. However, research on physical activity tends to focus on non-Indigenous youth, Westernized sports, exercise, and activities, as well as biomedical benefits rather than interconnected, holistic effects [2,3,4,30]. Lavallée [31] identified such studies as inadequate to encapsulate the unique physical activity experiences of Indigenous peoples and called for an Indigenous research framework to decolonize the often exploitative nature of research. Lavallée [31] used the medicine wheel to explore physical activity and sports among Indigenous people, which was a pioneering approach that highlighted the importance of incorporating Indigenous frameworks in programs for Indigenous people. In a later paper, Lavallée and Lévesque [32] presented an integrated Indigenous-ecological model, which bridges the strengths of Indigenous and Western knowledge, to guide physical activity programs for Indigenous youth. Reviews of physical activity of Indigenous youth also tend to broadly focus on mainstream sports and activities, and not specifically on traditional activities despite identifying youths’ deep connection to cultural physical activities [3,4]. Furthermore, as Paraschak and Thompson [33] suggest, a majority of approaches present physical activity among Indigenous peoples from a problem-based angle, rather than a strengths-based one. Distinctively, our systematic review employs the Lavallée and Lévesque [32] integrated Indigenous-ecological model to explore the holistic impacts of participating in traditional physical activities on Indigenous children and youth across the United States, Canada, New Zealand, and Australia. The field of traditional physical activity research is an emerging one, and more studies are needed to represent the complex ways Indigenous youth participate in these activities. Therefore, the aim of this systematic review is to explore the holistic and wellness impacts of traditional physical activity experiences among Indigenous youth and accumulate an evidence base for future research and programs.

## 2. Methods

### 2.1. Procedure

**Figure 1:**
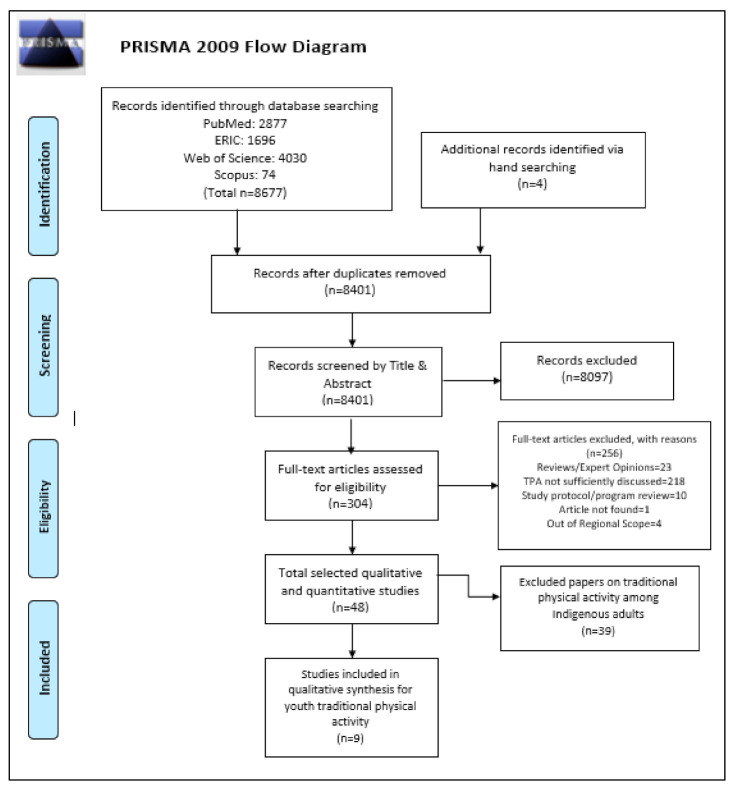
PRISMA flow chart of study selection, adapted from Liberati, Altman, Tetzlaff, Mulrow, Gøtzsche, Ioannidis, Clarke, Devereaux, Kleijnen and Moher [34].

The methods detailed below are reported based on the Preferred Reporting Items for Systematic Reviews and Meta-Analyses (PRISMA) guidelines [34]. Prior to starting the review, the authors, in consultation with a research librarian, agreed upon key words and MeSH terms. The search strategy included terms related to geographic locations, traditional physical activity (land based, hunting, gathering, etc.) and Indigenous populations (First Nations, Aboriginal, Inuit, Islander, etc.). A number of databases were selected based on their coverage of topics of interest. Pubmed and Web of Science were chosen for their coverage of medicine, science, and public health; and Education Resources Information Centre [35] was chosen for its coverage of education and school-based initiatives. Scopus was additionally searched to be inclusive of articles in the social sciences. A detailed record of the search strategy for each database can be found in Table S1. Due to differing filters for dates across databases, Table S1 also includes specific ranges used in each database to include articles between 2006 and 2018. A flow chart (Figure 1) depicting the search and screening process, was adapted from the PRISMA guidelines [34].

### 2.2. Inclusion and Exclusion Criteria

To be included in this review, articles needed to meet the following criteria. Peer-reviewed publications published online between January 2006 and May 2018 (the last search date) in the English language were included. The search period was chosen to be 10 years prior to the initial search in 2016, and repeated in 2018 (more details below). Geographically, only articles focusing on Indigenous populations in the United States of America, Canada, New Zealand, and Australia were considered due to similarities in colonial history and socio-political structures [36]. The articles broadly explored the experiences of children and youth participating in traditional physical activities employing at least one qualitative method (i.e., interviews, focus groups, etc.). Due to the expansive and complex definitions of what can entail “traditional physical activities” for youth [7], we attempted to be as inclusive as possible and relied on the primary studies’ identification of such activities. Studies where traditional physical activities are presented along with “mainstream” sports, exercises or activities were included if data from the relevant activities were able to be extracted. Noteworthy, we aimed to exclusively evaluate the perspectives of Indigenous youth as experts of their experiences. Children (ages below 14 years old) and youth (ages 15–24) were classified according to the definitions declared by the United Nations [37]. Articles that discussed or evaluated programs created for Indigenous youth but did not evaluate the experiences of youth via qualitative methods were excluded. Studies with multiple age groups were included if data from participants classified as children or youth were able to be extracted. Expert opinions, program reviews, literature reviews and reports published by governmental or non-governmental organizations were excluded. The flow diagram of study selection, adapted from Liberati, Altman, Tetzlaff, Mulrow, Gøtzsche, Ioannidis, Clarke, Devereaux, Kleijnen and Moher [34], detailing the selection process can be found in Figure 1.

### 2.3. Study Screening and Quality Appraisal

Following the aforementioned search strategy, search results from all four databases were combined and duplicates were removed. To be inclusive of a variety of diverse outcomes related to traditional physical activity, our search did not limit papers by specific health and wellness outcomes. Therefore, any articles within the selected timeframe reporting traditional physical activities, whether quantitative, qualitative, or mixed methods, for our populations of interest were selected for further appraisal. Articles were screened by their title and abstract and subsequently selected for full text review to assess their eligibility according to the inclusion criteria. The reference lists of the included articles were scanned for relevant papers that were missed via the original search. An independent reviewer conducted the search in 2016. The second search, repeated by a second reviewer with a similar but revised protocol, was conducted in 2018 to further ensure the inclusion of any relevant articles that were missed. Any disagreements with regard to the articles included were discussed by the authors until consensus was reached. Studies were critically appraised using a tool adapted from the Joanna Briggs Institute [38] Critical Appraisal Checklist for Qualitative Research. We assessed studies on several factors including strengths of study design, data analysis, findings, and degree of community partnership. The appraisal tool comprised 10 criteria (Table S2). The results of the appraisal were reviewed by authors, and conflicts were resolved after discussion.

### 2.4. Analysis

**Figure 2:**
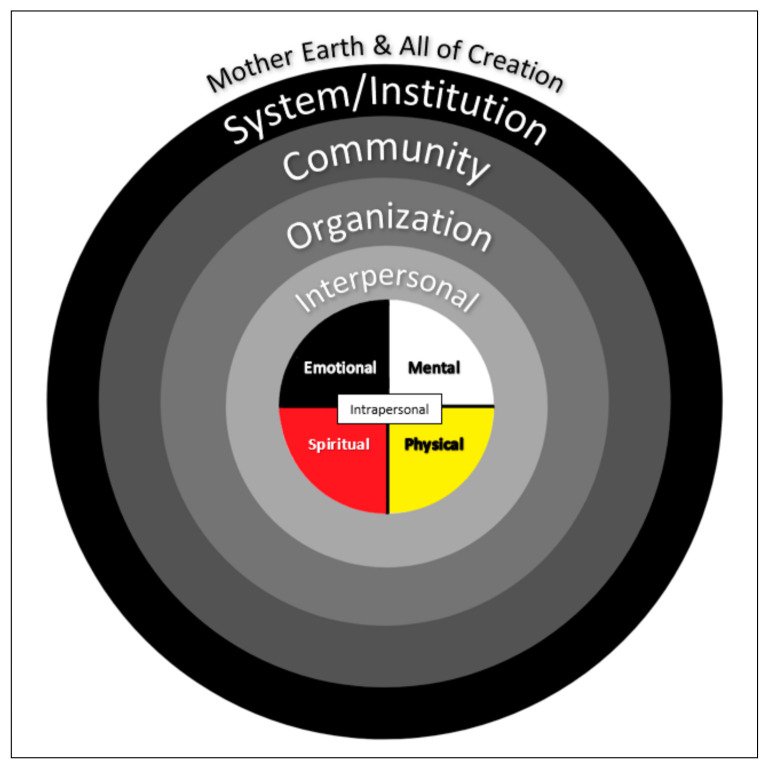
The Integrated Indigenous-ecological model, adapted from Lavallée [31] and Lavallée and Lévesque [32].

As settler scholars, we recognize the importance of synthesizing findings of this review within an Indigenous framework. To generate a rigorous and meaningful synthesis, we employed the integrated Indigenous-ecological model (Figure 2) as a framework to organize the findings across studies, which is consistent with other literature in the field [4,29]. The integrated Indigenous-ecological model was developed by Lavallée and Lévesque [32] and incorporates the social-ecological model of health behaviour proposed by McLeroy, et al. [39] with the teachings of the medicine wheel and Traditional Knowledge, and was specifically developed to explore sports experiences of Indigenous people. This model is guided by Mi’kmaq Elders Albert and Murdena Marshall’s two-eyed seeing approach (Etuaptmumk), which calls for enjoining the strengths of Indigenous ways of knowing with the strengths of Western ways [40]. This results in the analysis of traditional physical activity experiences of youth from various nested ecological viewpoints including intrapersonal (within person), interpersonal (between the individual and others), organizational, community, and policy (systemic levels). Importantly, it integrates the teachings of the medicine wheel, and allows for a deeper understanding of the interrelated, four-directional spiritual, emotional, mental, and physical (thus intrapersonal) impacts of participation in Indigenous activities (Figure 2). Within the medicine wheel, the mental relates to intellect, the emotional relates to feelings, the physical relates to the body, and the spiritual relates to the connection with the Creator, ancestors, land, nature and the four directional winds [32,41]. The four quadrants joining at the center represent the interconnectedness of health [32]. The socio-ecological model aligns with Indigenous perspectives of relatedness of self to the larger world. Inclusion of the medicine wheel within the framework incorporates connectivity to the land, which is an essential aspect of traditional activities, and considers delivery at each ecological level from a decolonizing approach [32]. Rather than viewing the impacts of physical activity as individual or micro-level effects, this approach allows for understanding the interconnectedness of larger macro-level systems affecting youth participation [32,40].

The synthesis was conducted by one reviewer (L.A.) and iteratively revised by two other reviewers (A.M.Z., L.J.S.T.). The results sections, along with relevant quotations, from each included study were repeatedly read, extracted, and analyzed. Direct quotes and interpretations by primary researchers were analyzed line by line and organized based on Dapice [41], Lavallée [31] and Lavallée and Lévesque [32] definitions of each realm within the medicine wheel, as well as the ecological levels. As each study was extracted, findings were organized into separate levels according to the aforementioned model to situate our findings in a culturally relevant, Indigenous research framework. Each step was conducted by one reviewer (L.A.), and revised by other reviewers (A.M.Z., L.J.S.T.).

## 3. Results

### 3.1. Study Selection and Appraisal

A total of nine articles were identified for inclusion in the review (Table 1). The PRISMA flowchart of the search and screening process can be found in Figure 1. Searching Pubmed (n = 2877), Web of Science (n = 4030), ERIC (n = 1696) and Scopus (n = 74) yielded 8677 articles in total to be screened by title and abstract. Search results from all four databases were combined and duplicates (n = 280) were removed. In total, 304 papers were selected subsequently for full text review to assess their eligibility according to the inclusion criteria. Of these, four studies were identified through reviewing reference lists and hand searching via Google Scholar. After full text review, a total of 48 studies were considered relevant to the broad topic of traditional physical activity. Of those, nine studies qualitatively evaluating the impacts of traditional physical activities on youth were included in this review. These articles were appraised according to an adapted version of the Joanna Briggs Institute [38] Critical Appraisal Checklist for Qualitative Research and assessed on 10 criteria (Table S2). Upon appraisal, a majority of studies were found to adequately fulfill each criterion. Notably, of nine studies, three studies specifically addressed their positionality as Western researchers [29,42,43]. Nevertheless, the remaining six were included as detailed accounts of community partnerships were mentioned. Additionally, two studies that do not explicitly identify participants who are adults older than 24 years were included as the participants were borderline for our age criteria [44,45]. Boyd and Braun [44] identify their age range as 18–25 (mean age = 20 years old), but do not state age for each participant in text. MacDonald, Willox, Ford, Shiwak, Wood, Government and Team [45] similarly identify the age range of participants as 15 to 25 years old.

### 3.2. Study Characteristics

The following characteristics are summarized in Table 1 and Table S3. Of the nine studies included, six were based on Indigenous youth in Canada [29,43,45,48,49,50], two in Australia [47,51], and one in the United States of America [44]. No articles on qualitative analysis of traditional physical activity of Indigenous youth in New Zealand were found. In total, 225 Indigenous youth participated in these studies. Three studies [29,44,45] included adult (older than 24 years) participants. Three studies employed focus groups as the method of data collection [44,47,50]. One study conducted one-on-one interviews along with talking circles [29]. Three studies employed semi-structured interviews [42,45,49]. One study conducted systemic observations [48] and another study used vignettes to collect youth experiences [43]. In addition to the above methods, two studies employed scales to measure some aspect of youths’ experiences [44,48], and two used supplementary stimuli (drawings, photographs) [42,50]. Of the nine articles, seven explicitly discussed partnerships with community members including Elders and/or experts in the development of study procedures [29,43,44,47,48,50], though all studies employed some degree of community involvement (Table S3).

### 3.3. Interconnected Experiences and Impacts

The experiences and impacts of participating in traditional physical activity are presented below situated in the integrated Indigenous-ecological model (Figure 2). Using this model, we represent the findings of the included studies nested in the following major areas: (a) intrapersonal (medicine wheel), (b) interpersonal, and (c) organizational, community and system.

#### 3.3.1. Intrapersonal

Numerous benefits, including spiritual, emotional, mental, and physical (Figure 2), are described by Indigenous youth when they participate in traditional physical activities. Youth were deeply connected and eager to participate in traditional physical activities, and described them as paths to experience spiritual growth [49] and be spiritually healthy [44]. A participant in Boyd and Braun [44] expressed enthusiasm for hula, saying, *“I just want to dance hula and keep on dancing.”* (p. 5). In Petrucka, Bassendowski, Goodwill, Wajunta, Yuzicappi, Yuzicappi, Hackett and Jeffery [43], youth mentioned, *“When we dance powwow we are at our healthiest… our choices are good and make us healthy.”* (p. 189). Youth in Crowe, Stanley, Probst and McMahon [47] similarly described enthusiasm for participating in cultural activities, including dances and survival skills, and children in the Pigford, Willows, Holt, Newton and Ball [50] study specifically identified cultural activities as healthy. Youth were passionate about traditional physical activity as it was associated with a strong sense of identity, pride for practicing their culture and thus their emotional health. In Crowe, Stanley, Probst and McMahon [47], youth described a sense of pride and self-esteem. Several children mentioned feeling “strong, happy, confident, safe, and special” when participating in traditional physical activities (p. 414) [47]. Youth in the Dubnewick, Hopper, Spence and McHugh [29] study mentioned feeling a sense of comfort and confidence when participating in Aboriginal games. They identified the importance of knowing and continuing Aboriginal games, mentioning, *“Some people might forget, so that is why you got to pass it on… every game has a meaning for what it does for us.”* (p. 214) [29]. When hunting and camping in Janelle, Laliberté and Ottawa [48], the researchers observed several positive mental and emotional impacts of traditional physical activities, including high motivation, sense of pride, and feeling supported. Furthermore, being on the land was described as a healing, nurturing, and stress-reducing experience that fostered good habits [45]. Participants in Nelson [42], Petrucka, Bassendowski, Goodwill, Wajunta, Yuzicappi, Yuzicappi, Hackett and Jeffery [43], MacDonald, Willox, Ford, Shiwak, Wood, Government and Team [45] specifically mentioned that traditional physical activities also kept youth busy, provided a sense of future direction, and prevented undesirable behaviours such as alcohol and substance abuse.

Youth in Kerpan and Humbert [49] expressed that these activities were a way to practice teachings, culture and identity, *“Culture is powwows and hoop dances, and how we grew up.”* (p. 1411). Furthermore, they strongly associated cultural physical activities as intrinsic to ‘Indian’ identity, as one youth mentioned, *“What sort of Indians would they be if they didn’t do that stuff!”* (p. 1411). One participant specifically stated a sense of happiness that they felt while sitting and watching powwow dances [49]. Similar notions of experiencing a strong cultural connection and pride were expressed in Nelson [42], MacDonald, Willox, Ford, Shiwak, Wood, Government and Team [45], Pigford, Willows, Holt, Newton and Ball [50]. An important aspect was the connection that youth felt to Elders when participating in traditional activities, which is identified as a positive factor for mental health within medicine wheel teachings [41]. Participants in Dubnewick, Hopper, Spence and McHugh [29], MacDonald, Willox, Ford, Shiwak, Wood, Government and Team [45], Crowe, Stanley, Probst and McMahon [47], Kerpan and Humbert [49] mentioned that activities such as fishing, hunting, and powwows were opportunities to connect with Elders and a means for sharing knowledge between generations. Similarly, participants in Dubnewick, Hopper, Spence and McHugh [29] mentioned that traditional physical activities were avenues for Elders to teach skills that are important for the bush and living on the land. Lastly, a few studies mentioned the physical benefits of participating in traditional physical activities. Traditional activities were associated with physical fitness and healthy eating. Youth described traditional activities as a means of strengthening and preparing their bodies for the bush [29]. Activities were also identified as physically demanding, but rewarding [49]. Cultural activities were also linked with food—many activities such as hunting, fishing, and powwows were connected to food procurement and communal eating, thus linking food and physical activity [47,50]. Nelson [42] described the physical benefits of these activities as “incidental” to the spiritual, emotional and mental benefits.

#### 3.3.2. Interpersonal

A majority of youth expressed traditional physical activities as group activities, and in general showed preference towards group experiences. Students in Boyd and Braun [44] wanted their school’s physical activity programs to be open to family, friends and community members. Traditional physical activities were described as familial and communal experiences across studies. When catching fish, a youth described how *“My dad guts it…and I cut it up”,* subsequently cooking it to share with the family (p. 413) [47]. Other youth described how their uncle took them swimming and spearfishing, mother taught them how to collect and eat oysters, and community members taught them dances [47]. In preparing for powwows, young women explained how their mother made outfits, and grandmother took them to ceremonies and round dances [49]. For male participants, male family members taught them how to hunt and fish [49], and took them to the bush to chop wood [45]. Participants in Pigford, Willows, Holt, Newton and Ball [50] also mentioned helping family members with community events and culture camps where they would also participate. Friends and family [43], and seeing Indigenous faces in general [29], provided a sense of safety and comfort for many participants. Youth in several studies also expressed a longing to pass their culture on to their own children, as one young mother explained, *“…I want to teach my girls too, so I want to pass on our culture because it’s something big... where [and] how I grew up”* (p. 136) [45]. A similar sentiment was expressed by a participant in Dubnewick, Hopper, Spence and McHugh [29] when discussing cultural continuity: *“Some people might forget, so that is why you got to pass it on…”* (p. 214).

#### 3.3.3. Organization, Community, and System

Though often implicit at macro-levels, the experiences of youth held some stories of barriers and promoters at organizational, community, and the larger social or systemic levels. Youth in Crowe, Stanley, Probst and McMahon [47] mentioned that their school did not offer enough opportunities for cultural activities in the curriculum, though programs, such as dance events and cooking, were available. Students in Boyd and Braun [44] expressed a similar interest in traditional activities incorporated within program offerings such as credit-bearing courses, with incentives such as tuition waivers. However, students expressed that these programs should be open to family and community members.

The impacts of community level factors were discussed by some youth. Community festivals were spaces for youth to present their cultural dances and sports with pride [42], and availability of local dance grounds had an impact on physical activity [47]. Youth in MacDonald, Willox, Ford, Shiwak, Wood, Government and Team [45] furthermore discussed that being on the land provided opportunities for them to connect as a community, feel secure and supported within their networks, and foster a sense of belonging.

Some discussions among youth in these studies were connected to larger socio-political and systemic factors, and several participants across studies mentioned “life before colonization”. Participants in Boyd and Braun [44] felt that their generation, as a whole, were less involved in Hawaiian culture due to Americanization and assimilation into mainstream culture. This meant less healthful activities and food consumption as a community, in comparison to daily living prior to Western contact—*“In the olden days they picked their food. They played all the sports… everything was off the land and ocean…”* (p. 5) [44]. Similar sentiments were expressed by youth in Dubnewick, Hopper, Spence and McHugh [29], who also felt that this was a reason to continue learning and passing on traditional games to future generations. Youth in MacDonald, Willox, Ford, Shiwak, Wood, Government and Team [45] mentioned, *“[Being on the land is] how people used to live before, so it’s important to still do those things sometimes.”* (p. 136) and found it important to connect to ancestors. 

## 4. Discussion

This systematic review explored the broad, holistic impacts of the traditional physical activity of Indigenous youth in North America, Australia, and New Zealand. We employed a culturally relevant integrated Indigenous-ecological model to illustrate youth experiences, and found several key experiences underpinning each (intrapersonal, interpersonal, organizational, community and systemic) level of the model [32]. Across studies, largely spiritual, emotional, mental, and some physical benefits of traditional physical activity were expressed by youth, which consistently highlighted the importance of participation in these activities. Youth described participation in these activities as holistically healthy, a way to experience connection to land, ancestry and community, and a means to develop healthy habits. Through exploring youth experiences, several underlying factors emerged that speak to the upstream factors impacting the micro-experiences of youth, and are critical to examine in tandem with intrapersonal lived experiences. Young Indigenous people expressed the need for physical activities to include friends and community members, which was similar to non-Indigenous youth [52]. Unlike non-Indigenous youth, they also mentioned that programs should be open to family members and described a sense of safety and comfort in seeing Indigenous faces. This could be attributed to the well-documented ostracization that Indigenous people experience in mainstream sports [53], as well as the historical uprooting of cultural practices due to colonization [8,9,11,12,13,14]. Traditional physical activities were described as essential group and family activities that exemplified community cohesion, which demonstrates their importance along with their social benefits. Descriptions of traditional activities were interweaved with familial and community interactions, a means for youth to learn from their parents, relatives, and Elders. Across studies, participants also expressed a strong interest in continuing traditions and understood the significance of passing on cultural practices. It is therefore essential that any programming incorporating traditional physical activities be inclusive of community and family members.

Consistent with previous discussions on Indigenous sports practices [7,33], traditional physical activities were also described as fluid, everyday experiences by participants across studies. Many Indigenous youth considered traditional activities to be part of daily living, which exemplifies the diverse definitions of physical activities for these youth [44,47]. Indigenous youth described traditional activities as physical activities that were a part of life and tradition, rather than exclusively categorizing them as exercise or fitness activities. The fluidity between food and physical activities is another significant factor to consider. Cultural activities such as powwows, hunting and fishing are related to both subsistence and exercise [47,50]. Therefore, participation in these traditional activities is not simply movement, but also nourishment. Participants in Boyd and Braun [44] stated spending time doing specific activities such as hula or canoeing won’t “pay the rent or bills.” Incorporating them into daily life and structures are primary ways of increasing participation. For students, a credit-bearing course, tuition waivers and educational incentives were attractive [44]. Integrating Indigenous activities into already existing Westernized sports-focused programs (i.e., school-based programs) was suggested as an avenue in some studies [47,50]. Yet, schools have historically and contemporarily been a place (and source) of systemic, colonial trauma and discrimination for Indigenous young people [54,55,56,57]. Therefore, we must question the extent to which traditional activities can be effectively participated in as a form of healing within a system that is inherently harmful.

The environment and community were factors that impacted the experiences of Indigenous youth. Community spaces were a quintessential aspect of traditional physical activities, and availability of resources and space were mentioned by some participants [45,47]. This is consistent with previous literature on Indigenous youth from various communities [46,58,59]. The external environment also includes educational institutions, where participants mentioned a lack of opportunities for traditional activities [44,47]. As discussed previously, future programming for Indigenous youth must critically consider the possibility of integration into existing school or university programs and curricula. Importance must also be placed on integrating community leadership in organizing such programs. Across studies in this review, youth repeatedly mentioned the significance of connecting with Elders as a key aspect of traditional activities. Previous literature have highlighted the generational harm when culturally inappropriate mentors or coaches are involved in programs created for Indigenous youth [60]. Involvement of Indigenous youth from communities as leaders in developing such opportunities is a further step to consider in cultivating ownership and agency for youth [43,60]. Notably, a majority of studies included in this review incorporated some level of community partnership when conducting their studies, though there were no methods consistently used by all studies. One aspect missing from the literature included in this review is a discussion of the often-distinct experiences of traditional physical activity that urban and rural Indigenous youth may have. As community settings can drastically change the kinds of barriers faced by youth, it is critical to explore and compare the differing experiences of rural and urban Indigenous participants in future research. Another missing aspect for further exploration is examining how anti-Indigenous racism and discrimination can affect youth participation in traditional activities [61].

Some gendered experiences were discussed by participants. Girls more frequently mentioned participating in traditional dancing [42,49] while boys were taught activities such as hunting, fishing, and chopping wood [45,47,49]. Like the distinction between rural and urban youth, gendered experiences were not explicitly explored in traditional physical activity literature. It is critical, however, to understand the experiences of young women compared to men in traditional activities, as there may be differences in activity choices, as well as factors such as self-esteem and shame which have been discussed as specific influences for Indigenous girls [62]. The necessity to study traditional activities from a decolonizing approach [31,32] is illustrated by youths’ longing for their ancestral life prior to colonial contact [29,44,45]. Though a shift to a “Westernized” lifestyle is not necessarily unique to Indigenous peoples, the distinction lies in their connection to land, ancestors, and nature, as well as the ongoing assimilation of Indigenous lives [32]. Rather than approach physical activity programming and research as individualistic, micro-experiences, youth called for the inclusion of cultural, familial, and communal connections. Lavallée and Lévesque [32] discuss the deficit-based Westernized approach when studying Indigenous physical activity experiences, where researchers often view them from a biomedical perspective. Thus, physical activities are sometimes viewed as preventative measures for potential antisocial or deviant behaviour rather than as holistic experiences. Interestingly, Indigenous youth in Petrucka, Bassendowski, Goodwill, Wajunta, Yuzicappi, Yuzicappi, Hackett and Jeffery [43], MacDonald, Willox, Ford, Shiwak, Wood, Government and Team [45] discussed how they viewed traditional activities as a means to stay busy and away from such behaviours. This may implicitly point towards the need for recreational opportunities within communities but, as Lavallée and Lévesque [32], Paraschak and Thompson [33] suggest, these opportunities must be created from Indigenous frameworks of balance and harmony, focusing on a strengths perspective, rather than as “treatment”. Approaching traditional activities from a decolonizing perspective, as mentioned previously, may not effectively occur within a system that necessitates the disenfranchisement of Indigenous youth. As youth discussed pre-colonial lifestyles, it is essential to examine the colonial pathways that continue to restrict Indigenous lives. Thus, decolonizing Indigenous experiences with traditional activities must be a product of systemic change—repatriating Indigenous land and upholding Indigenous sovereignty [35,63,64]. Though the literature studied in this review call for incorporation of traditional activities into existing programs (systems), ultimately, decolonization in action should go beyond settler-colonial programs or systems [64].

The findings in our review regarding the holistic benefits and barriers of traditional physical activities are consistent with previous reviews of sporting experiences of Indigenous youth [3,4,62]. However, our findings are a unique step forward in specifically studying the qualitative experiences of participating in traditional activities and outline a gap in research in this emerging field. Notably, though reviews of broad physical activity experiences among Indigenous youth found gendered and community specific differences discussed in the literature [3,62], our review identifies a need for deeper exploration of these areas in traditional physical activity research. Many studies excluded in our review also exclusively discussed the perspectives of program administrators, community members, parents, and Elders on youth physical activity, which are indeed essential viewpoints. However, further studies specifically incorporating youth voices and perspectives are necessary in order to achieve a full picture of youth experiences. Our review is the first of its kind to distinctively amalgamate traditional physical activity experiences of Indigenous youth using rigorous methodology, reported according to published guidelines. The findings in this review provide a wealth of knowledge on the effects of participation in traditional activities, can serve as a practical starting point for creating opportunities for Indigenous young people that is inclusive of their needs and interests, and identify gaps to address in future research. As Forsyth [65] calls for the necessity of an evidence base in Indigenous physical activities to guide future sports and recreational programs, this review provides a basis for the inclusion of traditional activities in such opportunities. Though our study employed rigorous methodology and included high-quality studies, there are some limitations. We strived to be inclusive of various health outcomes and traditional activities in our search strategy. However, it is possible that studies were missed due to indexing and differing classifications across databases, and our sample size (nine studies) is small. Many studies did not provide the specific questions asked in guiding their data collection; thus, themes may have emerged dependent on questions that were asked. A majority (six out of nine) studies focused on youth in Canada, and there were no articles representing youth in New Zealand. Selected articles also do not represent all Indigenous groups in our chosen geographic areas. Studies were also not equitable in ages included, as a majority focused on teens and young adults rather than young children, which is an issue across youth literature [66]. When adults were included in studies, generated data also tended to focus on adult perspectives more than youth. Lastly, we acknowledge that the experiences of Indigenous youth, who are diverse in community, culture, and circumstances, cannot be generalized or simplified, and that Indigenous knowledge is highly contextual [67]. This is particularly pertinent given the small number of studies, though rich in quality, included in this review. Rather, through our methods, we aimed to draw on shared experiences and themes across the literature, and consider the identification of these common threads as a strength of this review. Though this paper calls for the inclusion of traditional activities in programs for youth, we furthermore acknowledge that this may not be generalized for all Indigenous communities.

## 5. Conclusions

This review presented the health and wellness impacts of traditional physical activities on Indigenous youth situated in an integrated Indigenous-ecological framework. Through this systematic review, we identified the holistic benefits of traditional activities for youth, and the nature of the familial and communal relationships that affect these experiences. Furthermore, we explored the external, upstream factors that contribute as facilitators and barriers for traditional activity participation. Future studies are required to further understand the nuances of gender, discrimination, and regional contexts that may have an impact on traditional physical activities. In general, there is a scarcity of research qualitatively examining the perspectives of Indigenous youth experiences in traditional activities, and we identify this as a critical gap in forming an evidence base for inclusion of traditional physical activities in sports and physical activity programs. Ultimately, systemic change is necessary to decolonize the physical activity experiences of Indigenous peoples.

## Figures and Tables

**Table 1:** Characteristics of studies included in systematic review of the impacts of traditional physical activities (PA) on Indigenous youth.

Study	Research Question(s)/Purpose	Setting	Traditional Physical Activities Mentioned	Methodology	Sample Characteristics	Sampling Strategy	Data Collection Techniques
Boyd and Braun [44]	To understand the health perceptions, supports and barriers in planning prevention programs for Native Hawaiian college students	Native Hawaiian [46] community college students from Rural West O’ahu, Hawaii, USA	Paddling, hula, ancestral subsistence activities	Social marketing approach	n = 32 18–25 years old *	Research Assistants (RAs) visited courses with high NH enrollment at 4 community colleges, interested students told to contact	Focus group (FG) with closed circle seating Before FG, participants filled out 6-point scale ranging from very unhealthy to very healthy and provided info about how much exercise they do every week Asked 4 questions and solicited comments on 5 core prevention strategies to increase PA identified by the CDC and interest in course
Crowe, et al. [47]	(1) To explore the links between perspectives on culture, and healthy lifestyle behaviours for Indigenous Australian children’s (2) To explore approaching the development of intervention targeting lifestyle behaviours in Australian Indigenous children	3 urban towns in the south coast of New South Wales, Australia	Dancing, bush tucker walks, fishing, preparing foods	Not specified	n = 40 5–7 and 8–12 years old	Communities invited to participate in program, communities selected schools for participation purposive sampling via flyers and consent forms	FG with semi-structured discussion guide Attended by parent or Aboriginal Educational Officer
Dubnewick, Hopper, Spence and McHugh [29]	To understand how participation in traditional (Dene and Inuit) games can enhance the sport experiences of Indigenous youth	Northwest territories (NWT) communities, Canada	Traditional games	Community-based participatory research (CBPR)	n = 8 14–18 years old, and 10 adults*	Known-sponsor approach and purposeful selection—participants selected by regional sport and recreation representatives from the govt. of NWT who live in NWT communities	One-on-one interviews or talking circles
Janelle, et al. [48]	To increase self-esteem, re-establish cultural continuity, encourage pro-social (and prevent antisocial) behaviours among participants and ultimately empower and mobilize the community	Atikamek community of Manawan, Quebec Quebec, Canada	Hunting, trapping	Participatory observation	n = 6 14–17 years old	3rd author took young people from his community on 5 week stay	Systematic observations and adapted self-esteem scales (situational and dispositional)
Kerpan and Humbert [49]	To gather knowledge on the physical activity preferences and barriers of urban Aboriginal youth to develop culturally specific physical activity programming	Mid-sized prairie city, Canada	Dancing (jingle dancing, fancy dancing, jigging), hunting, fishing, hiking and drumming; powwow, round dancing	Ethnography, participatory methods	n = 15 Grades 9–12, 14–21 years old	Introduced project to students in classes, snowball sampling from initial participants	Semi-structured one-on-one interviews, informal conversations, participant observation
MacDonald, Willox, Ford, Shiwak, Wood, Government and Team [45]	To identify youth-specific protective factors that enhance well-being in the face of climate change, as well as how environmental change can challenge these factors	Nunatsiavut, Labrador, Canada	Being on the land, hunting, fishing, chopping wood	Case study	n = 17 15–25 years old *	Chosen by Local Research Coordinators	Semi structured interviews
Nelson [42]	To challenge assumptions about Indigenous young people, including “natural ability”, sport as panacea for health, education and behavioural issues	Independent urban school students, Australia	Dancing	Life Activity Project approach	n = 14 11–13 years old	Chosen based on questionnaire administered to all students in years 5–7; purposive selection	Semi-structured interviews with stimuli (drawings, photographs, diaries, maps)
Petrucka, Bassendowski, Goodwill, Wajunta, Yuzicappi, Yuzicappi, Hackett and Jeffery [43]	To determine the components of a “Living Well” initiative for youth participating in the “Positive Leadership, Legacy, Lifestyles, Attitudes, and Activities for Aboriginal Youth” (PL^3^A^3^Y) Project	Standing Buffalo First Nation, Saskatchewan, Canada	Powwow dancing, traditional games	Community-based participatory research (CBPR)	n = 78 11–13 years old	Students from elementary school who participated in culture camp	Vignettes on what they learned at culture camp
Pigford, et al. [50]	To explore First Nations children’s perceptions of food, activity, and health to inform a community-based prevention strategy	Rural plains Cree community, Alberta, Canada	Powwow dancing, hunting, playing on Treaty Day, ceremonial practices, playing at powwow grounds	Not specified	n = 15 8–10 years old	Study conducted at the request of community member, students in 4th and 5th grade eligible	FG employing storytelling and narrative—drawing pictures of activities

* Indicates articles with adult participants.

## Supplementary Materials

The following are available online at https://www.mdpi.com/1660-4601/17/21/8275/s1, Table S1: Search strategy for four databases (Pubmed, Web of Science, ERIC, and Scopus) included in systematic review for traditional physical activity of Indigenous youth; Table S2: Critical appraisal criteria for systematic review on traditional physical activity of Indigenous youth adapted from the Joanna Briggs Institute [38]; Table S3: Details on the framework of included studies, as well as analysis techniques and degree of community involvement for systematic review on traditional physical activity of Indigenous youth.
