# Supplementary material for: Health and Wellness Impacts of Traditional Physical Activity Experiences on Indigenous Youth: A Systematic Review
Source: Int J Environ Res Public Health. 2020 Nov 9;17(21):8275. doi: 10.3390/ijerph17218275 (PMC7664942; doi:10.3390/ijerph17218275)
Supplement: Supplementary file 1 [file ijerph-17-08275-s001.pdf]

International Journal of Environmental Research and Public Health

MDPI

Table 1. Search strategy for four databases (Pubmed, Web of Science, ERIC, Scopus) included in systematic review for traditional physical activity of Indigenous youth.

Search Strategy				
Names of database	Pubmed	Web of Science	ERIC	Scopus
Database time coverage	1900-Present	1900-Present	1966-Present	2004-Present
Date searched	April 25, 2016, May 13, 2018	April 26, 2016, May 12, 2018	April 27, 2016, May 11, 2018	May 14, 2018
Number of records	2877	4030	1696	74
Search strategy from each database	**(((((America* or Australia* or Canada or Canadian or New Zealand* or United States)) AND (aboriginal* or "first nation*" or indigenous* or inuit or islander* or métis or metis or native* or indian*)) AND (physical activity or activity or sport or exercise or fitness)) AND (subsistence or land or land-based or traditional or country or hunt* or gather* or fish* or forag* or cultur* or water))**	(ALL=(America* or Australia* or Canada or Canadian or New Zealand* or United States) AND ALL=(aboriginal* or "first nation*" or indigenous* or inuit or islander* or métis or metis or native* or indian*) AND ALL=(physical activity or activity or sport or exercise or fitness) AND ALL=(subsistence or land or land-based or traditional or country or hunt* or gather* or fish* or forag* or cultur* or water))	(america* or australia* or canada or canadian or new zealand* or united states) AND (aboriginal* or first nation* or indigenous* or inuit or islander* or métis or metis or native* or indian*) AND (physical activity or activity or sport or exercise or fitness) AND (subsistence or land or land-based or traditional or country or hunt* or gather* or fish* or forag* or cultur* or water)	TITLE-ABS-KEY (america* OR australia* OR canada OR canadian OR new AND zealand* OR united AND states) AND TITLE-ABS-KEY (aboriginal* OR first AND nation* OR indigenous* OR inuit OR islander* OR métis OR metis OR native* OR indian*) AND TITLE-ABS-KEY (physical AND activity OR activity OR sport OR exercise OR fitness) AND TITLE-ABS-KEY (subsistence OR land OR land-based OR traditional OR country OR hunt* OR gather* OR fish* OR forag* OR cultur* OR water)
Filters	Filters: **Humans, English, from 2006 - 2018** Sort by: **Publication Date**	*AND* **LANGUAGE:** (English) **Refined by: WEB OF SCIENCE CATEGORIES:** (SUBSTANCE ABUSE OR ENVIRONMENTAL SCIENCES OR AGRICULTURE MULTIDISCIPLINARY OR PUBLIC ENVIRONMENTAL OCCUPATIONAL HEALTH OR MULTIDISCIPLINARY SCIENCES OR PSYCHOLOGY MULTIDISCIPLINARY OR HOSPITALITY LEISURE SPORT TOURISM OR SOCIOLOGY OR BEHAVIORAL SCIENCES OR FISHERIES OR	Publication date: Last 5 years (in 2018); last 10 years (in 2016)	*PUBYEAR > 2005 AND PUBYEAR < 2019*

GEOGRAPHY OR SOCIAL SCIENCES BIOMEDICAL OR GEOSCIENCES MULTIDISCIPLINARY OR PSYCHOLOGY CLINICAL OR SPORT SCIENCES OR EDUCATION EDUCATIONAL RESEARCH OR ANTHROPOLOGY OR ENVIRONMENTAL STUDIES OR ORTHOPEDICS OR HISTORY OR NUTRITION DIETETICS OR HEALTH POLICY SERVICES OR REGIONAL URBAN PLANNING OR MEDICINE GENERAL INTERNAL OR NEUROSCIENCES OR HEALTH CARE SCIENCES SERVICES OR REHABILITATION OR INTEGRATIVE COMPLEMENTARY MEDICINE OR COMMUNICATION OR PHYSIOLOGY OR PSYCHIATRY OR CULTURAL STUDIES OR SOCIAL SCIENCES INTERDISCIPLINARY OR FORESTRY OR NURSING)

Timespan: 2006-2018. **Indexes:** SCI-EXPANDED, SSCI, A&HCI.

Adapted from: https://libraryguides.mcgill.ca/knowledge-syntheses/documenting

Table 2. Critical appraisal criteria for systematic review on traditional physical activity of Indigenous youth adapted from Joanna Briggs Institute [1].

Criteria		Boyd and Braun [2]	Crowe, et al. [3]	Dubnewick, et al. [4]	Janelle, et al. [5]	Kerpan and Humbert [6]	MacDonald, et al. [7]	Nelson [8]	Petrucka, et al. [9]	Pigford, et al. [10]
1.	Is there congruity between the stated philosophical perspective or study aim and the research methodology?	Yes	Yes	Yes	Yes	Yes	Yes	Yes	Yes	Yes
2.	Is there congruity between the research methodology and the research question or objectives?	Yes	Yes	Yes	Yes	Yes	Yes	Yes	Yes	Yes
3.	Is there congruity between the research methodology and the methods used to collect data?	Yes	Yes	Yes	Yes	Yes	Yes	Yes	Yes	Yes
4.	Is there congruity between the research methodology and the representation and analysis of data?	Yes	Yes	Yes	Yes	Yes	Yes	Yes	Yes	Yes
5.	Is there congruity between the research methodology and the interpretation of results?	Yes	Yes	Yes	Yes	Yes	Yes	Yes	Yes	Yes
6.	Is there a statement locating the researcher culturally or theoretically?	**No**	**No**	Yes	**No**	**No**	**No**	Yes	Yes	**No**
7.	Does the researcher adequately and appropriately discuss involvement of the community of interest in various stages of study?	Yes	Yes	Yes	Yes	Yes	Yes	Yes	Yes	Yes
8.	Are participants, their voices, and their communities adequately represented?	Yes	Yes	Yes	Yes	Yes	Yes	Yes	Yes	Yes
9.	Is the research ethical according to current criteria or, for recent studies, and is there evidence of ethical approval by an appropriate body?	Yes	Yes	Yes	Unclear (REB not mentioned)	Yes	Yes	Yes	Yes	Yes
10.	Do the conclusions drawn in the research report flow from the analysis, or interpretation, of the data?	Yes	Yes	Yes	Yes	Yes	Yes	Yes	Yes	Yes

Table 3. Details on included studies' framework, analysis techniques and degree of community involvement for systematic review on traditional physical activity of Indigenous youth.

Study	Framework	Data Analysis Techniques	Community Involvement
Boyd and Braun [2]	Not specified	Inductive transcript analysis → thematic analysis	Community action group (CAG) - Key informants included elders, college admins, college students, Native Hawaiians, health professionals. CAG guided procedures, feedback from participants was incorporated. CAG validated FG findings
Crowe, Stanley, Probst and McMahon [3]	Grounded theory	Thematic analysis	Community consultation to build trust/relationship, negotiation of objectives and procedures. Adjustments made on FG guide. Findings presented to community (Board of Managers)
Dubnewick, Hopper, Spence and McHugh [4]	Integrated Indigenous-ecological model	Inductive content analysis	Researchers had prior collaborating relationship with community. Research team included community members, negotiated purpose and procedure with communities. Elder and traditional game organization members provided feedback on FG findings
Janelle, Laliberté and Ottawa [5]	Not specified	Observation grid	Supervised by community workers, partnership with community teams to develop procedures and evaluation
Kerpan and Humbert [6]	Not specified	Ethnographical analysis	Researcher spent a year volunteering and coaching at the school prior to study. Triangulation of data and member checking with school and participants
MacDonald, Willox, Ford, Shiwak, Wood, Government and Team [7]	Not specified	Constant comparative method to develop concepts and codes	Results sharing workshops and open houses hosted in each community, validated themes and findings with participants, community members and LRCs
Nelson [8]	Critical race theory, post colonial theory	Thematic analysis	Author worked with school for 10 years prior, peer discussions
Petrucka, Bassendowski, Goodwill, Wajunta, Yuzicappi, Yuzicappi, Hackett and Jeffery [9]	Not specified	Thematic analysis	Youth and community were involved as co-researchers, researchers had long term relationships with Elders who contributed to study procedures. Knowledge sharing processes with participants and interested parties
Pigford, Willows, Holt, Newton and Ball [10]	Ecological lens	Thematic analysis	Study conducted by request of community member. Steering committee (Elders, educators, health workers, community departments) reviewed protocol and article, ensured cultural sensitivity. Children were included as key informants

References

1. Joanna Briggs Institute, JBI critical appraisal checklist for qualitative research. *http://joannabriggs. org/assets/docs/critical-appraisal-tools/JBI_Critical_Appraisal-Checklist_for_Qualitative_Research2017. pdf* **2017**.
2. Boyd, J. K.; Braun, K. L., Peer Reviewed: Supports for and Barriers to Healthy Living for Native Hawaiian Young Adults Enrolled in Community Colleges. *Preventing Chronic Disease* **2007,** 4, (4).
3. Crowe, R.; Stanley, R.; Probst, Y.; McMahon, A., Culture and healthy lifestyles: a qualitative exploration of the role of food and physical activity in three urban Australian Indigenous communities. *Australian and New Zealand journal of public health* **2017,** 41, (4), 411-416.
4. Dubnewick, M.; Hopper, T.; Spence, J. C.; McHugh, T.-L. F., "There's a cultural pride through our games": Enhancing the sport experiences of Indigenous youth in Canada through participation in traditional games. *Journal of Sport and Social Issues* **2018,** 42, (4), 207-226.
5. Janelle, A.; Laliberté, A.; Ottawa, U., Promoting traditions: An evaluation of a wilderness activity among First Nations of Canada. *Australasian Psychiatry* **2009,** 17, (1_suppl), S108-S111.
6. Kerpan, S.; Humbert, L., Playing together: The physical activity beliefs and behaviors of urban Aboriginal youth. *Journal of Physical Activity and Health* **2015,** 12, (10), 1409-1413.
7. MacDonald, J. P.; Willox, A. C.; Ford, J. D.; Shiwak, I.; Wood, M.; Government, R. I. C.; Team, I., Protective factors for mental health and well-being in a changing climate: Perspectives from Inuit youth in Nunatsiavut, Labrador. *Social Science & Medicine* **2015,** 141, 133-141.
8. Nelson, A., Sport, physical activity and urban Indigenous young people. *Australian Aboriginal Studies* **2009**, (2), 101.
9. Petrucka, P.; Bassendowski, S.; Goodwill, W.; Wajunta, C.; Yuzicappi, B.; Yuzicappi, L.; Hackett, P.; Jeffery, B., Positive leadership, legacy, lifestyles, attitudes, and activities for Aboriginal youth: A wise practices approach for positive Aboriginal youth futures. *International Journal of Indigenous Health* **2016,** 11, (1), 177.
10. Pigford, A.-A. E.; Willows, N. D.; Holt, N. L.; Newton, A. S.; Ball, G. D., Using First Nations children's perceptions of food and activity to inform an obesity prevention strategy. *Qualitative Health Research* **2012,** 22, (7), 986-996.
